# Supplementary material for: Are carbon emissions trading and green financial instruments synergistic? -Comprehensive quantitative research based on content analysis
Source: PLoS One. 2024 Mar 7;19(3):e0298601. doi: 10.1371/journal.pone.0298601 (PMC10919601; doi:10.1371/journal.pone.0298601)
Supplement: S1 Data — (ZIP) [file pone.0298601.s001.zip › Data/地级市Z_F工作报告.zip!/╣π╢1⁄2/╔╟═╖╩╨/└┤╫╘╣1⁄2╓┌║┼í╛┬φ┐╦╩2╛▌═°í┐_2018.docx]

各位代表：

我代表汕头市人民政府向大会作政府工作报告，请予审议，并请各位政协委员和其他列席人员提出意见。

一、2017年工作回顾

过去一年，市政府以习近平新时代中国特色社会主义思想为统领，在省委、省政府和市委的正确领导下，在市人大和市政协的监督支持下，深入贯彻党的十九大和省第十二次党代会、市第十一次党代会精神，按照“四个坚持、三个支撑、两个走在前列”的要求，抓重点、补短板、强弱项，着力破解长期制约汕头发展瓶颈，促进经济社会平稳健康发展，在加快实现汕头全面振兴、协调发展的征程中迈出坚实步伐。

（一）经济发展提速增效。预计全市地区生产总值2356亿元，增长9.2%。一般公共预算收入150.06亿元，增长11.2%。供给侧结构性改革不断深化，淘汰改造落后产能企业9家，消化房地产库存35.19万平方米，为企业降低成本40亿元。重点项目加速推进，39个省重点项目、277个市重点项目完成投资494亿元，带动固定资产投资完成2006亿元，增长27%。“12·28”签约、动工、竣工项目259个，投资总额2894亿元。消费潜力持续释放，全社会消费品零售总额1685亿元，增长11.2%。积极稳定外贸出口，进出口总额达577.8亿元，其中出口432.1亿元。

（二）扩容提质步伐加快。交通基础设施建设全面推进，内联外通快速交通网络加快形成。引进香港招商局开展战略合作，加快推进广澳港区建设。广梅汕铁路增建二线和厦深铁路联络线完成工程量87%，汕汕铁路、疏港铁路完成工可审查，城市轨道交通建设加快前期工作，汕头火车站换乘中心完成主体工程。揭惠高速南段建成通车，汕湛高速、潮汕环线汕头段全线铺开建设。海湾隧道开始掘进施工，大学路、磊口大桥续建全面完成，金凤高架桥建成通车，牛田洋快速通道、中山路东延、汕北大道（凤东路）启动建设，完成国省道改造92公里。新城建设初具规模。东海岸新城累计完成投资140亿元，东海岸大道全线通车，综合管廊启动建设。南滨新城潮汕历史文化博览中心主体结构建成，南滨路东段升级改造完工。旧城区修缮改造步伐加快。小公园历史文化街区完成首期14万平方米骑楼群外立面修复，修缮保育活化老妈宫戏台、彬园、开埠邮局陈列馆等项目，改造提升29家知名特色美食店。新建改建旅游厕所100座。乌桥岛棚户区改造启动土地房屋征收，安置区一期工程开工。

（三）产业升级成效明显。出台促进科技创新发展16条措施，启动创建国家创新型城市试点工作。新增国家级高新技术企业238家、省级工程技术研究中心42家、省级新型研发机构4家。新建科技企业孵化器5家、众创空间2家。全面推进全国质量强市示范城市、国家知识产权示范城市创建工作。汕头大学管轶团队荣获2017年度国家科学技术进步奖特等奖。濠江区获评“中国建筑之乡”，潮南区获评“全国服装（内衣家居服）产业知名品牌示范区”。金融业加快发展，新增金融主体9家，注册设立基金16家，设立广东华侨金融资产交易中心、大连商品交易所汕头塑料期货交割仓，新增A股上市企业6家、“新三板”挂牌企业9家、“华侨板”挂牌企业49家。“互联网+”新业态蓬勃发展，开通粤东首个跨境电商监管中心，淘宝镇、淘宝村数量列全省第一、第二位，连续三年进入全国“电商百佳城市”30强。三大战略平台取得重大进展。华侨经济文化合作试验区获批成为国家“双创”示范基地，泰盛科创园、明园科创金融城、潮商中心加快建设。汕头高新区升格为国家高新区，中以（汕头）科技创新合作区完成起步区规划修编和核心片区土地征收。临港经济区完成总体规划编制，广澳港区加快建设，建成汕头保税物流中心，启动比亚迪3D玻璃、上海电气风电装备项目，中海信（汕头）创新产业城完成首期招商。

（四）城乡面貌焕然一新。深入推进创文强管，获省文明办推荐参评全国文明城市提名资格。新建改建市政道路28条，完成中心城区市政道路沥青罩面169万平方米，道路通达能力明显提升。升级改造公园、绿地、道路绿化带30万平方米，收回整合原市面粉厂片区近200亩用地新建公园，城市建成区绿化覆盖率44.2%，呈现“四季常绿、季季有花”。大华路等沿街楼体外立面完成美化，老城街景重焕迷人光彩。实施以内海湾、时代广场为重点的城市景观照明工程，城市夜景更加绚丽多姿。拆除违章建筑物79.7万处，清理乱摆乱卖行为90.2万宗，查处交通违章行为66.3万宗，市容更加整洁有序。开展“百村示范、千村整治”美丽乡村建设三年大行动，基本完成96个美丽乡村示范村建设和252个行政村、214个社区整治，建成农村公路150公里，“村村通自来水”工程进展顺利，农村人居环境不断改善。生态环境治理扎实推进，练江水质持续向好，雷打石、潮南生活垃圾焚烧发电厂点火试运行。城市生活垃圾无害化处理率92.2%，城市污水集中处理率91%。城市空气质量优良率96.7%，PM2.5均值29微克/立方米。

（五）民生福祉显著改善。民生支出占公共财政支出超过75%。深入开展脱贫攻坚，完成相对贫困人口预脱贫4万人。新增城镇就业6.1万人、劳动力技能晋升培训2.6万人。教育事业均衡发展，公办、普惠性民办幼儿园占比提高到68%，推进县域内城乡义务教育一体化改革，高水平普及高中阶段教育，高考总上线率和中职毕业生就业率均达96%。广东以色列理工学院正式招生。金平区、龙湖区获省推进教育现代化先进区称号。推进创建卫生强市，新改扩建、异地建设医院18家，新增三甲医院1家、三级医院3家，成立医联体11个。扎实推进现代公共文化服务体系建设，新增省级非物质文化遗产项目10项、市级文保单位50个，广东潮剧院潮剧保护传承基地投入使用。实施樟林古港南粤古驿道保育活化。全民健身活动广泛开展，新增社区健身场地153个。举办“潮人杯”国际帆船赛、汕头国际半程马拉松赛，我市运动员在第十三届全运会上夺得3金、2银、4铜。养老服务体系逐步完善，提高普惠型高龄津贴，银龄安康行动统保率80.5%，养老床位达2.35万张。全面落实基本殡葬公共服务普惠政策。加大社保扩面征缴工作力度，城乡居民参加基本养老保险206.5万人、基本医疗保险502.3万人，参保率分别达100%和98%以上。农村留守儿童关爱服务体系基本建立。交通服务进一步优化，投放新能源公交车405辆，新建人行天桥2座，完成中心城区临时停车位规划，试点建设井筒式地下停车库。平安汕头建设扎实推进，全市刑事发案、火灾事故、交通事故致死人数分别下降8.9%、60%、8.9%。安全生产责任制全面落实，事故隐患排查治理力度加大，全市未发生较大以上生产安全事故。2017年确定的十件民生实事完成年度任务。

（六）政务环境持续优化。自觉接受人大、政协监督，办理人大议案、建议87件，政协提案182件，提请市人大常委会审议法规草案4件，制定、修改、废止政府规章8件。汕头基层社会组织联合会建设经验在全省推广。以作风建设年活动为总推手，政风建设成效明显，公职人员的服务意识、办事效率和队伍形象进一步提升。严格执行市直机关“三公”经费零增长。深化“一门式一网式”政府服务模式改革，市级43个部门895项行政许可和公共服务事项进驻网上办事大厅，实现“前台综合受理、后台分类审批、窗口统一出件”，完善区县及以下各级实体政务服务大厅建设，启用市行政服务中心24小时便民自助区。实施跨部门关联事项并联审批改革和重点项目审批代办制，取消、调整市级行政权责事项231项，清理规范中介服务事项63项。深化商事制度改革，实施“多证合一、一照一码”改革，新登记企业8989户、注册资本645.2亿元，分别增长21.6%、68.8%，市场主体总量突破30万户。口岸联检单位主动作为，通关效率大幅提升，在全省率先实现国际贸易“单一窗口”口岸、企业、报关、报检、舱单全覆盖，汕头口岸出口、进口通关速度分别列全国第三、第八位。国防动员、食品药品、计划生育、民族、宗教、港澳事务、对台、外事、侨务、残联、应急管理、信访、气象、人防、地震、统计、档案、科协、社科、地方志等各项工作取得新进展。

各位代表！过去一年，我们所取得的成绩和进步，是习近平新时代中国特色社会主义思想科学指引的结果，是省委、省政府和市委正确领导的结果，是全市人民同心协力、共同奋斗的结果，是海内外乡亲和各界朋友大力支持、积极参与的结果。在此，我代表市人民政府，向全市广大工人、农民、知识分子、干部职工，向给予我们支持的人大代表、政协委员、各民主党派、工商联、社会各界人士，向中直和省直驻汕单位，向驻汕解放军、武警官兵，向所有关心和支持汕头发展的港澳台同胞、海外侨胞和国际友人，表示崇高的敬意和衷心的感谢！

我们也清醒地认识到，工作中还存在很多不足，对标先进城市仍有较大差距，经济社会发展不平衡不充分问题亟待解决：一是实体经济面临的困难较多，企业融资难、用地难、用工难比较突出。二是产业结构调整任务艰巨，重大产业项目少，高技术制造业、先进制造业占比较低，新兴产业有待培育壮大。三是科技创新体系还不健全，全社会研发投入不足，创新型人才缺乏，科技孵化育成链条不完善，科技创新对产业的支撑作用不强，新旧动能转换有待加速。四是城乡环境治理任重道远，部分地区水环境污染严重，农村基础设施欠账多、环境脏乱差问题仍然存在，城市管理精细化水平有待提升，中心城区交通拥堵、交通秩序还没有根本改观。五是民生事业存在不少短板，医疗、教育、公共交通、文化体育等公共服务规模不足、水平不高，城乡区域资源配置不均衡。六是营商环境亟待优化，部分公职人员担当意识、改革意识不强，办事效率不高，群众和企业办事难问题仍然突出。对此，我们必须采取更加有力措施，切实加以解决。

二、2018年工作安排

今年是贯彻落实党的十九大精神的开局之年，是改革开放40周年，是决胜全面建成小康社会、实施“十三五”规划承上启下的关键一年。我们欣喜地看到，迈向新时代的汕头，正迎来全面振兴、协调发展的良好“起势”，一批批事关当前与长远发展的重大项目和重大平台正在紧锣密鼓推进，长期制约发展的交通、生态、服务效率等短板正在逐步补齐。尽管外部经济环境复杂严峻，区域竞争日趋激烈，我市在前进道路上仍面临诸多困难和挑战，但只要我们坚定信心，实干担当，奋勇争先，就一定能够走在广东决胜全面建成小康社会、加快建设社会主义现代化新征程前列。

今年政府工作的总体要求是：以习近平新时代中国特色社会主义思想为指导，全面贯彻落实党的十九大精神，按照中央经济工作会议、省委十二届二次、三次全会精神和市委十一届四次、五次全会的部署要求，坚持稳中求进工作总基调，坚持新发展理念，按照高质量发展的要求，全面学习对标赶超先进城市，深化供给侧结构性改革，深化创新驱动，进一步扩大开放，振兴实体经济，加强和改善民生，着力解决发展不平衡不充分问题，促进经济社会持续健康发展，加快打造与广东省域副中心城市定位相适应的现代化经济体系和城市发展新格局。

今年经济社会发展的预期目标是：地区生产总值增长9.5%以上，固定资产投资额增长20%以上，社会消费品零售总额增长10.5%以上，外贸进出口总额增长5%以上，一般公共预算收入增长8%以上，居民消费价格涨幅预期3%以内，常住居民人均可支配收入增长9.5%以上，城镇登记失业率控制在3%以内，节能减排降碳约束性指标完成省下达年度计划。

按照上述目标任务，重点抓好以下十方面工作：

（一）构筑“一湾一岸两河三平台”城市新格局，加快打造广东省域副中心城市。

按照《广东省沿海经济带综合发展规划（2017—2030年）》赋予汕头“省域副中心城市”定位的要求，深入贯彻市委关于构筑“一湾一岸两河三平台”城市新格局的战略部署，打造21世纪海上丝绸之路重要门户先行示范区，加快形成服务大汕头湾区的城市核心区，拉开国际化现代化大都市框架。

加快塑造湾区城市空间形态。坚持科学规划、有序建设、交通先行、产业先导、生态优先、产城融合，编制“一湾一岸两河三平台”建设实施纲要。按照“北优南拓，东融西联”的发展战略，以汕头内海湾为核心纵深拓展两翼，以东海岸新城为前沿吸纳聚集创新发展要素，以新津河、梅溪河为纽带构建特色生态景观廊道，以华侨试验区、国家高新区、临港经济区三大战略平台为载体，加快城市扩容提质，塑造“城在海边、海在城中”城市形态。汕头内海湾规划建设27.5公里的滨海走廊，依托珠港新城和南滨新城，打造潮商总部区和休闲商务区，带动牛田洋、三屿围片区和榕江生态经济带规划建设，形成两岸互济、城景相融的活力内湾区；东海岸新城瞄准国际标杆，建设粤东中央商务区，打造国际化湾区新城、现代化创新都市，塑造最美黄金海岸；新津河沿线统筹城镇、村庄、景区建设，突出自然风光和人文历史的融合，布局中央水廊道公园带，建设大地景观和人居环境诗意共融的生态绿色走廊；梅溪河沿线聚焦风貌保存、生态修复、城市更新，活化历史文化遗存，增加滨河开放空间，打造城市景观文化再生走廊。

统筹推进三大战略平台建设。突出三大战略平台的引领和载体作用，强化产业、人口和创新要素导入，打造生产、生活、生态同步发展的产城融合示范区。华侨试验区发挥华侨华人资源优势，紧扣合作、创新、服务主题，构建汕头经济特区创新引领区、粤东现代产业发展高地和中国华侨经济文化合作平台。以华侨未来城为龙头，推进中泰（汕头）华侨中心、泰盛科创园、明园科创金融城、潮商中心等项目建设。国家高新区实施“政区合一”，整合政策和空间资源，打造自主创新核心区和国际科技合作承载区。加快高新区扩区和中以（汕头）科技创新合作区建设，推动天安数码城（汕头）产业城建设。临港经济区按照“前港中区后城”的模式，构建港产城联动发展的滨海城市格局。建设外海港区，坚持区港联动，统筹保税区、濠江区、潮阳海门镇的规划建设。发挥“保税区+保税物流中心”优势，打造集出口加工、商品交易、物流配送、跨境电商、服务贸易等功能于一体的对外贸易中心。大力发展临港装备制造业，构建现代临港产业带。

引领促进区域协调发展。发挥城市核心区的示范引领作用，东融澄海、南澳，西联潮阳、潮南，加快全市域一体化、紧凑型发展。澄海区加快城区扩容提质，高起点规划建设东部沿海片区，加快形成与东海岸新城联动发展的城市组团。南澳县着力发展全域旅游，创建国家5A级旅游景区，建设生态文明海岛。潮阳区加快建设城市副中心，着力打造榕江南岸片区城市景观带和新经济走廊。潮南区加快建设承担区域中心服务职能的新城区，提升跨区域竞争力。推进汕潮揭同城化，加快汕潮揭临港空铁经济合作区建设，提升高端产业创新集聚、交通网络互联互通、公共服务同城共享、生态环境保护合作水平，携手建设一体化融合发展的汕潮揭特色城市群。

（二）推进“一一三五六六”工程建设，全力打造全国性综合交通枢纽。

统筹推进全市交通一体化，在本届政府任期内建成一个大港口、一大批市政道路、三条跨江海通道、五条铁路、六条市政大通道，实现六条高速公路互联互通，形成比较完整的大交通格局，增强汕头作为省域副中心城市的集聚辐射功能。

加快建设港口“头号工程”。广澳港区二期工程建成两个10万吨级集装箱码头泊位和10万吨级航道，力争开工建设三期工程，完成前澳埠村整体搬迁。发挥香港招商局整合资源优势，推进“智慧港口”建设，完善港口配套服务，继续实施扶持政策，多开班轮航线，拓展货源腹地，促进港口货物吞吐量大幅增长。

加快改造一大批市政道路。完成长江路、汕樟路、新兴路等一批道路升级改造，启动嵩山路、衡山路、珠池路、东厦路、金湖路等新一批道路升级改造，继续实施中心城区道路加铺沥青罩面。推进泰山路、黄河路快速化改造，在中心城区核心区谋划快速通道，形成快速内环。推进泰山路、珠池路、外马路、中山西路、金砂西路、金砂中路、招商路、民族路、跃进路和2018汕头国际马拉松赛沿线等路段建筑立面美化工程。

加快建设三条跨江海通道。加快汕头海湾隧道、潮汕环线跨榕江特大桥建设，全面启动牛田洋快速通道建设。三条跨江海通道建成后，将构建中心城区快速外环线网，全方位打通汕头湾南北交通主动脉，加速推进南滨新城、牛田洋、三屿围、榕江片区开发建设。

加快建设五条铁路。广梅汕铁路增建二线和厦深铁路联络线建成通车，汕头火车站换乘中心同步投入使用。启动汕汕铁路、疏港铁路建设，形成与珠三角2小时交通圈。推进城市轨道交通建设规划报批和1号线前期工作，完成云轨试验段建设。

加快推动六条高速公路互联互通。揭惠高速全线建成通车，加快汕湛高速、潮汕环线建设，与沈海、汕昆、潮惠高速互联互通，构筑汕头与珠三角的快速路网，形成市域内半小时、汕潮揭1小时交通圈。

加快建设六条市政大通道。金砂路西延、中山路东延、汕北大道（凤东路）加快建设，金砂西路及金砂中路升级改造、金鸿公路市政化改造、海滨路东延一期工程启动建设。六条市政大通道建成后，将引领中心城区“东融西联”，支撑城市扩容提质。

此外，加快一批普通干线公路建设。按照“交通+旅游”发展新模式，加快省滨海旅游公路汕头示范段规划建设。完成国道324线红旗岭路段升级改造，启动省道232线护堤路、省道234线关埠至金灶段、省道236线陈沙大道等升级改造，推进汕南大道濠江段等干线公路项目建设，启动南澳连接汕汾高速联络线前期工作。

（三）坚持产业兴市，加快构建区域现代产业高地。

产业兴则汕头兴，产业强则汕头强。坚持以供给侧结构性改革为主线，推动经济发展质量变革、效率变革、动力变革，加快新旧动能转换，促进产业高端化发展。

支持实体经济发展。坚持暖企惠企护企，深入开展企业大走访大调研，全面落实省“实体经济十条”和市的实施意见，全力降低制造业企业生产成本和制度性交易成本。着力解决融资难，推进国家产融合作试点城市建设，发展股权投资基金，扩大信贷风险补偿资金和政策性担保普惠面，发放中小微企业服务券，用活融资租赁、商业保理和互联网小贷新兴金融工具，探索供应链金融和普惠金融服务，争取新增境内外上市企业和“新三板”挂牌企业15家以上。着力解决用地难，完善现代产业差别化、精细化用地政策，实施弹性年期出让、先租后让等多种土地有偿使用机制。着力解决用工难，完善用工引智服务政策，提高就业供需匹配度，强化公共就业服务。加快国有企业改革重组发展步伐，优化国资布局结构，推动国有企业做强做大，确保国有资产保值增值。加大企业培育力度，净增规模以上工业企业80家以上，加快培育一批年主营收入超100亿元、300亿元的龙头企业。

推动园区提质增效。编制全市工业园区专项规划，以产城融合理念，构建园区发展新格局。划定工业用地保护红线，至2020年全市工业控制用地不少于80平方公里并严控改变用途。推广产业集聚发展模式，支持发展楼宇经济，开展标准厂房建设试点。完善产业转移园区“4+3”布局，推进金平现代产业集聚区、龙湖东部新兴产业园、南山湾科技产业园建设。培育主导产业，引导集聚发展，努力打造一批年产值超500亿元、1000亿元产业集群。

提升招商引资实效。围绕“大平台、大产业、大项目、大企业”，开展靶向招商、产业链招商，积极引进产业龙头企业和大数据、互联网综合服务提供商，实现“签约一批、动工一批、投产一批、储备一批”。做好跟踪服务，确保“12·28”重点项目加快落地、开花结果。

推进现代产业发展。大力培育发展新兴产业。推动高端智能装备、新材料、电子信息、生物医药等产业发展壮大。加快推进比亚迪跨座式单轨和3D玻璃、上海电气风电装备等项目建设。加快传统产业优化提升，全面落实“中国制造2025”行动计划，支持企业加快数字化、网络化、智能化改造，支持中小企业设备更新和骨干企业扩产增效，打造一批轻工包装、工艺玩具、内衣服装等行业龙头企业。加快发展现代服务业，全年服务业增加值占地区生产总值比重达46%以上。大力实施“引金入汕”，构建更为完善的金融服务体系。加快与腾讯、华为等信息产业龙头企业合作，发展互联网、大数据、云计算现代信息服务业。发展众创、众包、众扶、众筹等新业态、新模式。推进现代供应链发展，建设华润万象城、万达广场、粤东绿地中心、红星美凯龙城市综合体和粤东天环冷链、广澳港物流园。建设工业设计中心，大力发展工业创意产业，举办第二届“市长杯”工业设计大赛。加快建设南澳岛、内海湾、小公园等重点旅游区域，大力发展滨海、都市、乡村旅游。

全面建设质量强市。大力实施名牌带动战略和技术标准战略，以质量提升带动产业升级。推进国家洗涤护肤用品质检中心、省轻工机械产业计量测试中心等公共技术服务平台建设，完善公共检测技术服务体系。推动潮阳、潮南创建“广东省质量强市（县、区）示范区”，澄海创建“全国动漫产业（玩具）知名品牌示范区”，濠江创建“全国滨海潮汕休闲旅游知名品牌示范区”，力争创建全国质量强市示范城市通过省预验收并申报国家验收。

（四）深入实施创新驱动发展战略，建设国家创新型城市。

创新是引领发展的第一动力。汕头实施创新驱动发展有基础、有需求，要优化区域创新生态，着力推动创新经济再升级，建设国家创新型城市和区域科创中心。

培育一大批创新能力强的龙头企业。持续推动高新技术企业精准培育工作，新增国家级高新技术企业180家以上。支持现有高新技术企业加强核心技术攻关，发展成为科技“小巨人”企业，全年高技术制造业增加值增长15%以上。提升企业研发创新能力，新增国家和省市重点实验室、工程实验室、工程研究中心、企业技术中心等创新载体120家以上，规模以上工业企业研发机构覆盖率达25%以上。

打造科技创新成果转化孵化育成链条。加快重大创新平台建设。龙湖工业园区力争获批省级高新区。整合资源申报组建省实验室。培育省级临床医学研究中心，鼓励名校名院在汕设立创新研究院，推进汕头机器人与智能制造研究院、“一带一路”科技创新与服务研究院等研发机构建设，打造一批生产性服务业公共服务平台。实施科技创新孵化育成机构树标提质行动，加快发挥中海信等孵化育成机构效应，探索建立异地孵化器。大力发展创业投资，与省粤科金融集团等知名机构合作，建立政府引导型创业投资基金。主动对接国内外优质创新资源，鼓励设立股权投资母基金，推动风险投资与创新项目常态化对接，引导社会资本投向战略性新兴产业、优势传统产业。引进高水平创新创业服务机构，举办更具影响力、更具实效的创新创业大赛，吸引参赛优质项目和创新团队落地汕头。构建资金链、技术链、产业链、政策链四链融合发展的创新创业生态。

健全科技创新政府扶持机制。落实科技创新发展16条等政策，探索多元化的投入机制，构建良好的区域创新生态。加大政府和社会科技投入，全年R&D经费投入增长35%以上。扩大科技企业信贷风险补偿金受益面，运用信贷贴息、担保及保险补助等手段，完善全链条科技金融服务体系。促进产学研深度融合，建设博士和博士后创新创业孵化基地、理工类研究生科技创新实践基地。全面落实加快人才发展实施意见，发挥海内外人才工作联络站、“海智”基地工作站作用，实施高端人才引进“金凤计划”。强化知识产权创造、保护和运用，推进重点领域专利保护试点和重点企业保护直通车工作，支持中国（汕头）玩具快速维权中心高效发挥作用，实施知识产权优势企业培育工程，争创国家知识产权示范城市。

（五）推进新时代改革开放，持续优化营商环境。

营商环境是一个城市的软实力，也是核心竞争力。必须对汕头营商环境进行“革命性再造”，全面深化重点领域和关键环节改革，加快构建开放型经济新体制。

营造便捷高效政务环境。实施打造国际化法治化便利化营商环境对标厦门行动计划，力争今年营商环境达到或超过厦门市水平。以“数字政府”建设为突破口，推动政务信息系统整合共享和数据开放，不断提升“一门式一网式”政府服务和网上办事大厅办事效率，进一步推进行政许可审批事项和公共服务事项进驻各级行政服务中心。深入推进“互联网+政务服务”，实现“一门在基层、服务在网上”。加强12345政府服务热线建设，完善工作机制，提高服务能力。

构建公平竞争市场环境。全面实施公平竞争审查制度，加快升级统一开放、竞争有序的市场体系。深化商事制度改革，全面推进企业登记全程电子化。推进市场准入负面清单制度和企业投资项目准入负面清单管理改革，推动市场准入便利化，保障内外资企业平等权益。实行守信联合激励和失信联合惩戒制度，强化事中事后监管，建立以信用为核心的市场监管体系。推进综合行政执法，实现“双随机、一公开”监管全覆盖。加强和完善全市统一的公共资源交易平台体系建设，着力推进公共资源交易信息化、规范化、透明化。

推动形成全面开放新格局。发挥经济特区先行先试优势、华侨试验区特色优势和保税区功能优势，大胆开展基础性和核心制度创新，争取进入国家自由贸易试验区行列。加快汕头保税物流中心封关运营，形成“保税+国际贸易”“保税+现代物流”“保税+跨境电商”新业态。发挥华侨试验区引侨聚侨作用，加快“侨梦苑”建设，推动设立华侨产业母基金。优化出口产业结构，引导传统产业向高附加值环节延伸，扶持优质外贸企业做大做强。鼓励发展外贸新业态新模式，大力发展跨境电商和服务贸易。加大力度推进大宗商品进口。继续推进“单一窗口”建设，打造一流的口岸通关服务环境。拓展与“一带一路”沿线国家多领域合作，推进中欧区域政策合作试点工作。

（六）深入推进创文强管，提升城市文明水平。

以全国文明城市创建标准作为“指挥棒”，各区（县）、各相关职能部门要坚持“一盘棋”思想，把社会主义核心价值观贯穿于创文强管全过程，对标开展工作，强化城市管理，提高城市净化、序化、亮化、美化水平，不断提升市民文明素养和城市文明程度。

大力整治市容环境，净化提标。持续开展主次干道、公共场所、城乡结合部、背街小巷等重点部位市容“六乱”专项整治行动，进一步规范集贸市场管理，落实“门前三包”，解决市容市貌突出问题。推进“厕所革命”，新建、升级改造一批公共厕所，推动全市公共厕所在数量、质量和功能配套上实现大提升。实施环卫保洁巩固提升工程，强化主次干道路面、人行道清洗保洁和降尘工作，推进城乡环卫作业一体化，完善市、区、街道三级环卫作业质量检查考核体系，逐步推行垃圾分类减量工作。加快数字化城市管理建设，实现中心城区市政、园林、环卫、城管执法、道路停车等管理全覆盖。

实施交通改善工程，序化提档。优先发展公共交通，建设科学、便利、快捷的公交体系。建设长平路—华山路—中山路首期快速公交系统，新增建设4个公交站场，新增投放新能源公交车600辆以上，提高公交线网覆盖面和班线发车密度，确保城市公共交通分担率达25%以上，切实解决市民出行问题。规范共享单车管理。规划建设一批人行天桥、隧道。充分利用公共绿地、广场、学校操场、住宅小区等建设地下停车场、立体停车库，首批井筒式地下停车库建成运营，运用智慧停车管理，破解停车难、停车乱难题。继续推进文明交通行动计划，开展礼让斑马线活动，严厉查处酒驾醉驾、机动车闯红灯、逆行等严重交通违法行为，以海滨路禁摩为突破口，积极稳妥推进中心城区“限摩”，不断完善道路交通安全管理设施，加大警力投入，推进智慧交通系统建设，实现道路交通秩序明显改观。

推进景观照明建设，亮化提品。加快编制汕头市中心城区城市景观照明规划，健全市区景观亮化管理工作机制。建立智慧照明监管平台，完善城市照明系统设施建设，形成以内海湾两岸为核心，主要商圈、城市主要出入口、标志性建筑物为重点，重要道路和高架桥为脉络的城市夜景节能灯光体系。实现主干道装灯率100%，亮灯率99%以上；街巷道路装灯率100%，亮灯率95%以上。

提升城市形象品位，美化提质。加快推进小公园片区保育活化，启动“三环三线”二期工程建设，全面完成开埠区核心区骑楼群修缮。全力推进乌桥岛棚户区改造工作，三年内完成6700户棚改任务。开工建设新津河、梅溪河景观工程。提升城市园林绿化建设品位和管理水平，启动实施中心城区绿化提升行动，继续推进一批公园绿地改造，完成中心城区高速出入口绿化美化，提升城市花化彩化水平。

涵养市民文明素质，内化提升。广泛开展社会风尚提升行动，持续开展文明交通、文明旅游、文明乡风、和谐邻里等教育活动。举办“汕头道德讲堂”“书香飘万家”等主题实践活动，开展道德模范、“汕头好人”等评选活动，壮大向上向善的社会正能量。大力弘扬志愿服务精神。深入推进文明村镇、文明单位、文明家庭、文明校园等群众性创建活动，加强和改进未成年人思想道德建设，推动社会文明进步。

（七）坚持生态优先，建设美丽汕头。

牢固树立绿水青山就是金山银山的发展理念，着力解决城乡突出环境问题，不断满足人民群众日益增长的优美生态环境需要，加快建设天蓝、地绿、水清的美丽汕头。

强化环境污染治理。打好水、大气、土壤污染防治攻坚战。全面落实“河长制”，推进“五水共治”，实施“一河一策”。突出练江流域综合整治，推进练江水闸、海门湾水闸、垃圾焚烧发电厂、纺织印染环保综合处理中心、污水处理厂及配套管网等基础设施建设；扩大练江流域畜禽禁养区范围，坚决清空禁养区内各类畜禽养殖场；严厉打击环境违法行为，重点查处造纸、印染、电镀等重污染企业违法行为。加快榕江流域汕头段水环境综合整治，推进韩江、榕江、练江三江连通工程，加强韩江流域联防联治。加快推进黄厝围沟、鮀济河等黑臭水体整治。全面实施江河海水面保洁，确保城市集中式饮用水源100%达标。完善中心城区西片区截污管网，启动西区污水处理厂建设。推进大气环境治理，巩固提升空气环境质量。实施土壤污染防治行动计划，开展重点行业企业用地土壤污染状况调查、土壤污染治理与修复试点。巩固提升贵屿电子拆解业污染整治成果。

筑牢生态安全屏障。加强山水林田湖草生态保护，做好岸线、湿地、水体等生态保护修复工作。积极创建“国家森林城市”，建设碳汇林15960亩，生态景观林带10公里，森林覆盖率达30.45%，城市建成区绿化覆盖率达44.5%。加强海洋综合管控，保护海洋生态环境，推进南澳岛蓝色海湾整治及南澳岛国家级海洋牧场示范区、青澳湾国家级海洋公园建设。

推进节能减排降耗。深化国家节水型城市创建。支持绿色清洁生产，推广节能产品和技术，推动园区循环化改造。强化机动车环保监管，加快充电基础设施建设和新能源汽车推广应用。推广绿色建筑和装配式建筑，实施公共建筑节能改造，综合利用建筑废弃物。在全社会倡导绿色生产生活方式。

（八）实施乡村振兴战略，加快推进农业农村现代化。

坚持农业农村优先发展，科学制定乡村振兴战略规划，加快建设产业兴旺、生态宜居、乡风文明、治理有效、生活富裕的美丽乡村。

坚决打赢脱贫攻坚战。健全稳定脱贫长效机制，继续落实好产业扶贫、就业扶贫、金融扶贫，以及低保和教育、医疗、住房“三保障”扶贫惠民政策。注重扶贫同扶志、扶智相结合，激发贫困人口内生动力。加强考核监督，完成2.4万贫困人口预脱贫目标，37个省定贫困村提前建成社会主义新农村示范村。

深化农业农村改革。全面完成农村土地承包经营权确权登记颁证工作。稳妥推进农村集体产权制度改革，抓好澄海区国家农村集体产权制度改革试点县试点工作。推动农村集体“三资”管理规范化、制度化、信息化、长效化。大力发展农村普惠金融，扩大政策性农业保险。推进农业供给侧结构性改革，大力发展特色农业、精品农业和效益农业，保障优质安全农产品有效供给。培育新型农业经营主体，发展农业龙头企业、农民合作社、家庭农场。培育农业新产业、新业态，支持发展休闲农业与乡村旅游业，建设一批广东农业公园和农业与乡村旅游示范镇（点）；培育农产品加工和农村、农业电商示范企业，完善农产品流通经营服务网络，促进农村一二三产业融合发展。全面深化供销合作社综合改革，加快建设汕头供销社光华茶博园。加快南澳后江渔港、达濠渔港建设。推进粮食市场化改革，完善粮食安全保障体系。

加快美丽乡村建设。推动创文强管向农村延伸，深入开展“百村示范、千村整治”美丽乡村建设三年大行动，完成348个村居环境整治，建设100个美丽乡村示范村。突出“三清三拆”、露天粪坑、畜禽养殖整治，解决农村环境“脏乱差”问题。深入开展“九个一建设”，强力补齐村道建设、一体化供水、垃圾转运处理、生活污水处理等短板。注重突出潮汕特色，保护古建筑、古村落等历史文化资源。推进中小河流治理和高效节水灌溉建设，达标加固建设一批江海堤围。实施粤东灌区续建配套与节水改造工程，推进“百河千沟万渠”大整治行动，完成潮阳、潮南引韩供水工程，完成“村村通自来水”工程建设。加快推进“四好农村路”建设，完善300人以上自然村通硬化公路建设，实现全市建制村通客运班车100%目标。

（九）切实优化公共服务供给，不断增强人民获得感幸福感。

坚持以人民为中心的发展思想，努力满足人民日益增长的美好生活需要，让改革发展成果更多更公平地惠及全市人民。

千方百计促进就业和增收。实施更加积极的就业创业政策，加快人力资源服务产业园建设。全面加强职业技能培训，新增城镇就业5.2万人、劳动力技能晋升培训2.55万人。实施差别化收入分配激励政策，稳步调整企业职工最低工资标准，促进收入分配更合理、更有序。

推动教育优质均衡发展。深入推进教育现代化，完成濠江区、潮阳区、潮南区教育现代化先进区申报工作。发挥公办幼儿园示范效应，普惠性民办幼儿园、规范化幼儿园覆盖率分别达到80%和60%。统筹推进县域内城乡义务教育一体化改革发展，继续实施“初中学校品质提升三年行动计划”，着力消除义务教育阶段择校热、大班额现象。稳妥推进高中阶段学校考试招生制度改革试点工作，推动一批公办高中学校达到省一级以上办学标准，鼓励普通高中特色发展。推进职业教育集团化办学、产教融合、中高职衔接，加快汕头卫生健康学院、汕头民用航空培训学院、汕头学前教育学院筹建步伐，创建省现代职业教育综合改革示范市。继续支持汕头大学、广东以色列理工学院特色办学，启动广以二期校区建设。加快推进金平、龙湖、潮南特殊教育学校改扩建、新建工作。发展继续教育和社区教育。支持和规范社会力量兴办教育。加强师德师风建设，培养高素质教师队伍。推进区县中小学教师“县管校聘”管理体制改革，启动实施乡村小学全科教师培养计划。

加快健康汕头建设。创建卫生强市，打造粤东医疗高地。加快推进市中医院、市妇幼保健院、市皮肤医院等医院异地扩建，力争潮南民生医院、潮阳大峰医院通过“三甲”评审。支持和规范社会办医，加快潮阳白求恩医院、宜华华侨医院、国瑞医院等民营医院建设。升级改造20家区县级医院，完成15家乡镇卫生院标准化改造，建设第二批公建民营规范化村卫生站，建成全市基层医疗卫生信息化平台。深化公立医院改革，加快医联体建设。整合建立统一的基本医疗保险制度，深化医保支付改革。实施家庭医生签约服务扩面工程，确保签约率达到30%以上，重点人群达到60%以上。大力发展中医药事业和健康产业。加强医德建设，构建和谐医患关系。加强重点传染病防控。确保通过“国家卫生城市”复审。深入实施“全面两孩”政策，推进公共场所母婴设施建设，提高计划生育服务管理水平。

完善多层次社会保障体系。全面实施全民参保计划，强化社会保险扩面征缴，落实企业养老保险省级统筹。加大底线民生保障力度，提高城乡低保补差水平和特困人员供养标准，全面建立困难残疾人生活补贴和重度残疾人护理补贴制度。推进市社会福利中心、市救助安置中心建设，升级改造市救助管理站，启用市慈爱托养中心和市慈爱康复中心，健全农村留守儿童、困境未成年人和空巢老人关爱体系，推广适度普惠型儿童福利制度，继续完善高龄老人政府津贴制度，探索建立失能人员长期照护制度，支持社会力量兴办养老机构。加快建立多主体供应、多渠道保障、租购并举的住房制度，加强保障性安居工程建设。完善社会救助标准自然增长机制，发展社会福利、社会慈善事业。

促进文化事业文化产业繁荣发展。完善公共文化设施，建设公共数字文化服务网络，打造常态化文化惠民活动平台，基本实现行政村（社区）综合性文化服务中心全覆盖。加强对革命文化、华侨文化和潮汕优秀传统文化的保护、宣传和推介，加强文物保护和非遗保护传承，推进小公园专题博物馆群、市档案馆新馆建设，做好潮汕历史文化博览中心公益性展馆布展工作。发展新闻出版事业，繁荣文化艺术精品创作，举办系列高雅艺术和群众性文化演出活动。扩大对外文化交流合作，推动潮汕文化“走出去”。加大文化产业园区扶持力度，鼓励发展文化创意产业。加强“扫黄打非”和版权保护工作，确保文化领域安全。加快体育强市建设，广泛开展全民健身活动，提升竞技体育实力。积极申办2022年第十六届省运会，全力筹办2018汕头国际马拉松赛，办好“潮人杯”国际帆船赛、全国跳水冠军赛、全国帆板锦标赛等赛事，展示汕头新形象。

继续办好十件民生实事。计划投入资金23.1亿元，集中力量办好十件民生实事。一是加大教育投入。二是提高困难群众救助标准。三是提升城乡医疗卫生服务水平。四是提高城乡居民基础养老金标准。五是改善农村生活生产环境。六是加强城乡环境治理。七是升级改造一批中心城区道路。八是改善困难群众居住条件。九是继续实施小公园开埠区改造修复。十是实施公交惠民工程。

（十）提升社会治理水平，建设和谐平安汕头。

坚持总体国家安全观，全力打造共建共治共享的社会治理格局，进一步增强人民群众安全感。

维护社会大局稳定。加强和改进信访维稳工作，积极防范化解社会矛盾。落实意识形态工作责任制。完善反恐维稳和应急处突工作机制，严防暴力恐怖、宗教极端活动发生。依法打击“黄赌毒黑拐骗”等重点违法犯罪行为，坚决惩治严重暴力犯罪。全面推进全民禁毒工程。加大对涉盗抢、涉假、涉税、涉枪、涉“三非”外国人等问题整治力度。加大金融监管力度，防范金融风险。

推进公共安全体系建设。扎实推进“雪亮工程”，加快公共安全视频系统和反恐维稳指挥中心规划建设，不断健全立体化治安防控体系。加强公安处突队伍建设，健全快速反应机制。严格落实安全生产“党政同责、一岗双责、齐抓共管、失职追责”责任体系和企业主体责任，建立完善消防安全责任制体系，深化生产安全事故隐患排查治理，严防较大以上生产安全事故。加强食品药品安全监管，推进检验检测机构建设，加大专项整治和联合执法力度，让人民群众吃得安心、用得放心。推进应急管理基础性工作，完善各类突发事件应急预案，加强“双盲”应急演练。加强地质灾害监测、台风预警响应等工作，全面提升防灾减灾救灾能力。

夯实基层治理根基。加强社会治理体系建设，推动社会治理重心向基层下移，实现政府治理和社会调节、居民自治良性互动。完善城乡社区治理，推进城乡网格化管理，加强城乡社区协商，健全基层民主自治机制。完善基层社会组织联合会建设，引导规范基层社会组织参与社会治理、发挥积极作用。实施“社会组织+”行动计划，支持和规范社会组织承接政府购买服务，实施社工“双百五年行动计划”，推进社工人才队伍建设。

与此同时，积极推进军民融合深度发展，做好国防动员、双拥优抚工作，巩固军政军民团结。继续深化殡葬改革。做好民族、宗教、港澳事务、对台、外事、侨务、人防、地震、气象、统计、档案、科协、社科、地方志等各项工作。

各位代表！做好政府今年各项工作，必须牢固树立政治意识、大局意识、核心意识、看齐意识，坚决维护以习近平同志为核心的党中央权威，坚持党的领导，抓好市委各项决策部署的落实，全面加强政府自身建设，努力开创政府工作新局面。一是加强民主法治建设。自觉接受市人大及其常委会的法律监督、工作监督和市政协的民主监督，认真办理人大议案建议和政协提案，广泛听取民主党派、工商联、无党派人士和工会、共青团、妇联等群团组织的意见建议。二是全面加强依法行政。加快法治政府建设，强化依法行政考评。健全依法决策机制，提升政府决策法治化水平，推进严格规范公正文明执法。深入开展“七五普法”，加强司法行政工作。三是强化行政权力监督。完善政府内部层级监督，健全行政权力监督机制。全面深化政务公开，自觉接受社会监督和舆论监督。四是建设廉洁政府。认真落实全面从严治党主体责任，切实加强政府系统党风廉政建设和反腐败工作。深入推进作风建设，严格落实中央八项规定及其实施细则，驰而不息正风肃纪，坚决纠正“四风”问题。切实加强队伍建设，强化廉政教育，完善监督机制，打造信念过硬、政治过硬、责任过硬、能力过硬、作风过硬的公务员队伍，树立风清气正的政府形象。

各位代表！“时代是出卷人，我们是答卷人，人民是阅卷人！”新时代的号角已经吹响。新的一年，让我们更加紧密地团结在以习近平同志为核心的党中央周围，在省委、省政府和市委的坚强领导下，团结带领全市人民，凝聚海内外潮人和社会各界的智慧和力量，锐意进取，只争朝夕，推动汕头加快全面振兴、协调发展，以优异成绩迎接改革开放40周年，向人民交出满意的答卷！
